# Selective Retention of Bone Marrow Stromal Cells with Gelatin Sponge for Repair of Intervertebral Disc Defects after Microendoscopic Discectomy: A Prospective Controlled Study and 2-Year Follow-Up

**DOI:** 10.1155/2021/4822383

**Published:** 2021-07-13

**Authors:** Baoshan Xu, Hao Zhang, Lilong Du, Qiuming Yuan, Kaihui Zhang, Haiwei Xu, Xinlong Ma, Yue Liu, Hongfeng Jiang, Ning Li

**Affiliations:** ^1^Department of Minimally Invasive Spine Surgery, Tianjin Hospital, Tianjin University, Tianjin 300211, China; ^2^Graduated School of Tianjin Medical University, Tianjin 300070, China; ^3^Tianjin Medical University General Hospital Airport Hospital, Tianjin Medical University, Tianjin 300308, China; ^4^Baodi Hospital, Tianjin Medical University, Tianjin 301800, China

## Abstract

**Objective:**

Discectomy remains the classic procedure for treating lumbar intervertebral disc (IVD) herniation, but the occurrence of defects after discectomy is thought to be an important cause generating recurrent and accelerated IVD degeneration. Previous studies attempted suture of the annulus fissure, but the validity of this technique on restraining the degenerative process is controversial. On the other hand, cell therapies have been shown in multiple clinical and basic studies. Our purpose was to investigate the effectiveness of selective retention of autologous Bone Marrow Stromal Cells (BMSCs) with gelatin sponge in combination with annulus fibrosus suture (AFS) for the repair of IVD defects following mobile microendoscopic discectomy (MMED).

**Methods:**

This prospective, two-armed, and controlled clinical study was conducted from December 2016 to December 2018. Written informed consent was obtained from each patient. Forty-five patients with typical symptoms, positive signs of radiculopathy, and obvious lumbar disc herniation observed by MRI were enrolled. Patients were divided into 3 groups with different treating methods: MMED (*n* = 15), MMED+AFS (*n* = 15), and MMED+AFS+BMSCs (*n* = 15). A postoperative 2-year follow-up was performed to evaluate the patient-reported outcomes of VAS, ODI, and SF-36. The improvement rate of VAS and ODI was calculated as [(latest‐preoperative)/preoperative] to evaluate the therapeutic effect of the three groups. Assessment parameters included Pfirrmann grade, intervertebral disc height (IDH), and disc protrusion size (DPS), as measured by MRI to evaluate the morphological changes.

**Results:**

All patients enrolled had a postoperative follow-up at 3, 6, 12, and 24 months. VAS and ODI scores were significantly improved compared to the preoperative status in all three groups with a mean DPS reduction rate over 50%. At the final follow-up, the improvement rate of the VAS score in the MMED+AFS+BMSCs group was significantly higher than the MMED+AFS and MMED groups (80.1% ± 7.6% vs. 71.3% ± 7.0% vs. 70.1% ± 7.8%), while ODI improvement showed a significant change (65.6% ± 8.8% vs. 59.9% ± 5.5% vs. 57.8% ± 8.1%). All participants showed significant improvement in SF-36 PCS and MCS; the differences between each group were not significant. The mean IDH loss rate of the MMED+AFS+BMSCs group was also significantly lower than other groups (−17.2% ± 1.3% vs. −27.6% ± 0.7% vs. −29.3% ± 2.2%). The Pfirrmann grade was aggravated in the MMED and MMED+AFS groups while maintained at the preoperative grade in the MMED+AFS+BMSCs group. No adverse events of cell transplantation or recurrence were found in all patients during the postoperative follow-up period.

**Conclusions:**

It is feasible and effective to repair lumbar IVD defects using SCR-enriched BMSCs with gelatin sponges, which warrants further study and development as a cell-based therapy for IVD repair.

## 1. Introduction

Lumbar intervertebral disc (IVD) herniation is a common disease which mainly results in lower back pain, radiculopathy and disability, generating high medical expenses and social burden. Fortunately, through multiple conservative treatments, nearly 90% of the patients achieve reliable relief, but for the remaining 10%, surgical therapy seems to be an irreplaceable choice [1]. Discectomy is a frequently used surgical procedure for IVD herniation treatment, and its validity has been proven in numerous studies, but 20%-25% of patients still had poor results [2, 3]. The IVD defect generated by discectomy leads to further IVD degeneration and disc herniation recurrence or intervertebral instability, which may lead to intractable chronic pain or numbness that often requires additional surgery.

Several attempts have been made clinically to seal IVD defects by sutures or rigid buttress devices. Bailey et al. used an FDA-approved X-close device to suture annulus fibrosus (AF) defect after discectomy [4]. In addition, commercial annular closure devices such as Barricaid implant and Inclose Mesh were used to block off the IVD defect by forming a mechanical barrier [5, 6]. However, these repair techniques focus on closing the wound of the defect without functional restoration of the IVD, and some scholars found that the additional annulus fibrosus suture (AFS) had no significant superiority [4, 7]. Therefore, a biological repairing method is needed to induce IVD repair.

IVD are avascular structures lacking nutritional supply, which creates a major challenge for self-repair [8]. IVD degeneration may be regarded as a metabolic disorder originating from decreased IVD cell quantity and quality, resulting in a massive loss of extracellular matrix (ECM) and a hypoxic, acidic microenvironment [9–12]. Cell therapy is a promising reparative strategy, especially mesenchymal stem cells (MSCs) with the efficacy of rescuing and reactivating nucleus pulposus cells isolated from degenerated discs by enhancing ECM expression and synthesis [13–18]. In recent years, cell therapy with culture-expanded MSCs has been reported in the clinical treatment of IVD degeneration, which provides pain relief and functional recovery and significant symptom relief and shows a remarkable potential to repair IVD degeneration [19, 20].

However, most of the studies used culture-expanded MSCs for transplantation, with the risk of differentiating abnormality and tumorigenicity, which is time-consuming, has complex steps, and is difficult for clinical application. Bone marrow aspirate (BMA) is considered an important source of MSCs in both clinical and experimental studies. BMA has been cleared by the FDA and represents a possible biological option for use in the treatment of musculoskeletal disease [21–24]. Unfortunately, the MSCs represent a very small fraction of about 0.001-0.01% of nucleated cell count of the BMA [25–27], leading to a limited therapeutic effect. Selective cell retention (SCR) technology could concentrate target cells (including stem and progenitor cells) and effective components from BMA into a carrier material to facilitate the rapid attachment of nucleated cells [26, 28–30]. Gelatin sponge consisting of multiple types of collagen is a common hemostatic material that is widely used in clinical treatment, which is multiporous in structure and absorbable that could be an ideal scaffold material. Our pilot study has shown that after the process of SCR enrichment of BMA, the adhesion folds of nucleated cells and target cells are 6.40 ± 0.93 and 4.20 ± 0.65, respectively. Moreover, enriched BMSCs were cultured *in vitro* for 11 days, and many colony-forming units were stained by crystal violet in the culture dish, which revealed that the enrichment procedure could effectively retain the cell validity (Supplemental material Figs. S1 and S2). Hence, SCR technology for enriching graft materials, without the need for ex vivo expansion, holds great potential for intraoperative cell preparation.

Lumbar discectomy was usually performed using minimally invasive mobile microendoscopic discectomy (MMED, Storz, Germany) in our department, and the AF defect was usually sutured when possible; our purpose was to investigate the efficacy of IVD repair using autologous selective retention of Bone Marrow Stromal Cells (BMSCs) combined with gelatin sponge and AF suture for repairing IVD defect after MMED surgery. Our secondary aim was to evaluate the validity of AF suture alone after discectomy.

## 2. Methods and Materials

### 2.1. Study Design

**Figure 1:**
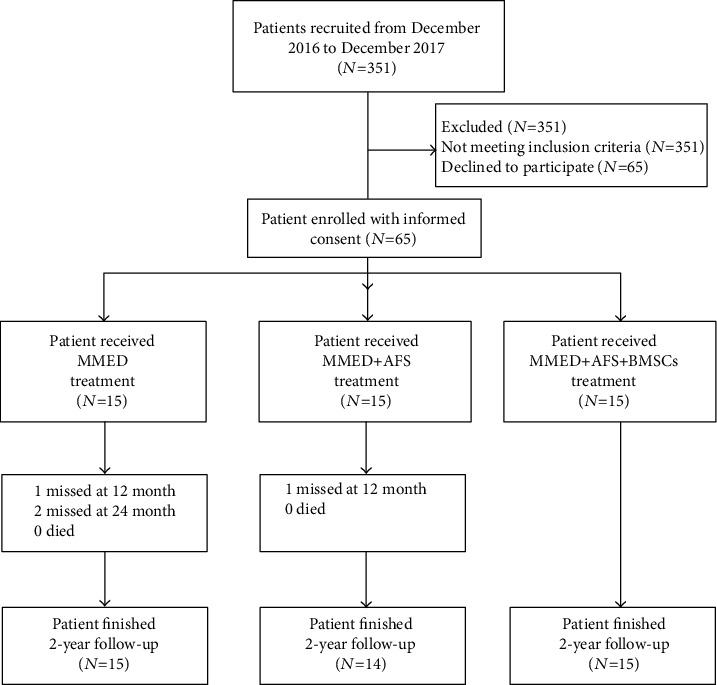
A flow chart shows the process of patient enrollment.

Patients experiencing symptomatic lumbar disc herniation were enrolled from a single center (Tianjin Hospital) from December 2016 to December 2017, with imaging confirmation of single-level disc herniation. Patients were randomly divided into 3 groups with different treatment strategies: (1) MMED group: patients were treated with MMED alone; (2) MMED+AFS group: the AF defect was sutured by a Disposable Annular Stapler after the MMED process; (3) MMED+AFS+BMSCs group: after MMED, enriched autologous BMSC-seeded gelatin sponge was inserted into the IVD defect under MMED before AF defect was sutured (Figure 1).

Our study was a prospective, two-arm, and controlled clinical study, conducted in accordance with the Declaration of Helsinki (Ethical Principles for Medical Research Involving Human Subjects), and has been registered at ClinicalTrials.gov (NCT03002207). The study was approved by the ethics committees of Tianjin Hospital (2016001).

### 2.2. Inclusion and Exclusion Criteria

Inclusion criteria of the patients are the following: (1) participants with typical symptoms, accompanied by positive signs of radiculopathy based on physical examination; (2) failed conventional treatments (physical and medical) for at least 3 months; (3) subjects with soft disc herniation at level 1, which was demonstrated on CT and MRI; and (4) participants who signed informed consent of cell transplantation before treatment.

Exclusion criteria are the following: (1) participants with incomplete medical records; (2) evidence of infection, osteochondrosis at the responsible segment, severe lumbar spinal stenosis based on MRI, or previous history of lumbar spine surgery; (3) participants receiving persistent anticoagulation therapy; (4) uncontrolled dementia and/or inability to sign informed consent; (5) MRI contraindication (e.g., cerebral aneurysm clips, cochlear implants, pacemaker, and biostimulators); (6) pregnancy; and (7) other serious systemic diseases such as autoimmune diseases, hematopoietic diseases, or malignant tumor.

### 2.3. Interventions

#### 2.3.1. Surgical Procedures

Patients were placed in the prone position on the operating table under general anesthesia. The position and direction of the target intervertebral space were located with C-arm fluoroscopy. After routine sterilization and draping process, a 2 cm longitudinal incision was made lateral to the spinal process. Subcutaneous tissue and deep fascia were incised, and the paraspinal muscle was distracted from the spinous process and lamina; then, the working (outer) tube was inserted. Once the inferior border of the proximal lamina was exposed under direct vision, the operating (inner) tube was immediately inserted followed by an endoscope imaging system. A fenestration was then created with the removal of the partial inferior border of the lamina and inferior articular process. The nerve was retracted and protected using cotton pieces or a retractor, and the herniated disc was fully exposed. A short incision was made at the apex of the herniation, and degenerated disc material between the intervertebral space was then removed using nucleus pulposus (NP) forceps. Finally, the disc space was rinsed with sterile saline to flush out residual NP tissues.

#### 2.3.2. Annulus Fibrosus Suture (AFS)

In the MMED+AFS group, the defect was sutured using an EFit Disposable Annular Stapler (2020 Medical Technology Co. Ltd, China). Briefly, the annulus fissure was sufficiently exposed, and then, the straight needle was inserted into the annulus fibrosus lateral to the annulus fissure. According to the operating procedures of Disposable Annular Stapler, we threaded the suture through the annulus fissure on both sides, pulled it out, and knotted it. And then, the extra sutures were cut off after confirming that the sutures were reliable, and the nerve decompression was effective. In the MMED group, the defect after discectomy was left untreated.

#### 2.3.3. BMA Harvest and Enrichment of Autologous BMSCs

**Figure 2:**
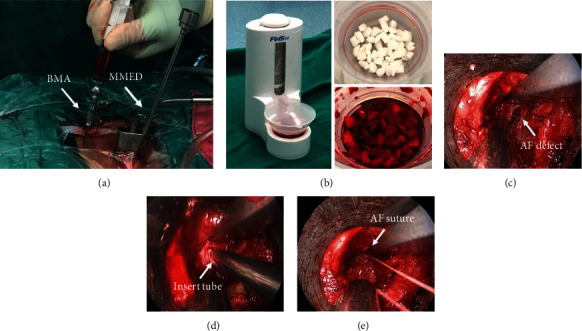
A unilateral posterior superior iliac crest puncture was performed; a total of 30 mL of BMA was harvested (a). Then, BONE GROWTH PROMOTER (B1) was used to filter and enrich BMSCs into the gelatin sponge cubes (B2); after 6 cycles of enrichment, the composite was fully prepared for implantation (B3). The composite was inserted into the AF defect (c) using an insert tube (d). After the implantation procedure, AF was sutured using a Disposable Annular Stapler (e).

The area of the posterior superior iliac spine (PSIS) was sterilized during MMED. Normal saline (2 mL) and heparin (1 mL, 6250 units) were prepared and drawn into a 50 mL syringe. Then, a PSIS puncture was performed; as soon as the cancellous bone layer was reached, a total of 30 mL BMA was harvested. One piece of gelatin sponge (60 mm × 20 mm × 5 mm; Xiangen Medical Technology Development Co. Ltd, China) was cut into 5 × 5 mm cubes and then layered into a BONE GROWTH PROMOTER (FUWOSI, Chongqing, China), which was used as a SCR device. The SCR process was performed using negative pressure to facilitate the filtration of the BMA through the biomaterial [22]. Five cycles were processed until the active ingredients were fully filtered, and target cells were captured by the gelatin sponge cubes; the composite was then prepared for implantation (Figures 2(a) and 2(b)). The time of the whole procedure was approximately 5 minutes.

In the MMED+AFS+BMSCs group, 10 pieces of BMSC-seeded gelatin sponge cubes were inserted into the disc space after discectomy. After the disc space was sufficiently filled with graft materials, AF defects were sutured immediately (Figures 2(c)–2(e)). A thin drainage was placed, and the incision was subsequently closed.

#### 2.3.4. Postoperative Management

Routine antibiotic infusion was implemented at the intraoperative period and postoperative day 1 with the drainage removed within 12-24 h. Patients were encouraged to do moderate lumbar muscle exercises off-bed with gentle waist movements after removal of drainage. After discharging from the hospital, patients were suggested to carry out regular rehabilitation training.

### 2.4. Follow-Up and Outcome Measures

A 2-year postoperative follow-up was performed. The protocol included 5 visits that were, respectively, carried out in preoperative and postoperative (3, 6, 12, and 24 months). Patient self-reported evaluation scales and MRI imaging parameters, and data were collected and collated by a single physician.

#### 2.4.1. Visual Analogue Scale (VAS)

VAS scores were used to evaluate the intensity of pain. Patients were asked to give a subjective estimate of their pain on a scale of 0-10. A base of 0 meant no pain; 1-3 scores represented mild pain that did not significantly affect daily life; a score of 4-6 represented medium pain that affected sleep; a score of 7-10 represented severe pain that could not be tolerated, and a score of 10 represented extreme and debilitating pain. The change in VAS was recorded at each visit in terms of [(latest‐preoperative)/preoperative].

#### 2.4.2. Oswestry Disability Index (ODI)

The ODI assessed the level of disability based on a 10-question questionnaire, which included pain intensity, personal care, lifting, walking, sitting, standing, sleeping, sex life, social life, and traveling. Each question had 6 choices and was represented by a scale of 0-5, where a higher score meant more serious disability. Patient self-reported scores in the proportion of the total points were defined as the ODI. The change in ODI at each visit was calculated according to [(latest‐preoperative)/preoperative].

#### 2.4.3. Short Form-36 (SF-36) Life Quality Questionnaire

The SF-36 questionnaire was widely used in the assessment of the life quality of patients and evaluation of clinical interferences, giving a comprehensive description that included 2 aspects: a physical score and a mental score. Each aspect was divided into four parts, including short questionnaires with quantifiable answers. Each of the four parts was expressed as a percentage of the total points, where a higher percentage represented better physical and mental conditions.

#### 2.4.4. Imaging Index

All patients underwent MRI scans preoperative and 12 months postoperative. Pfirrmann classification was used to evaluate the severity of IVD degeneration based on T2-weighted images [31]. Intervertebral disc height (IDH) and disc protrusion size (DPS) were measured according to the method reported by Smuck et al. [32]. IDH loss rates and DPS reduction rates were calculated according to [(latest‐preoperative)/preoperative].

### 2.5. Statistical Analysis

SPSS v22 (IBM, Armonk, NY software) was used for statistical analysis, and data were represented as the mean ± standard deviation (S.D.). One-way analysis of variance (ANOVA) was performed on VAS, ODI, and SF-36 scores before surgery and 3 months, 6 months, 12 months, and 24 months after surgery. The VAS and ODI improvement rate and imaging index analysis between different groups were compared by Student's *t*-test. *P* < 0.05 was considered statistically significant.

## 3. Results

### 3.1. Patient Demographics

351 patients were recruited from a single center (Tianjin Hospital) from December 2016 to December 2017. 45 patients who fit the inclusion criteria (28 males and 17 females; 43 ± 9.8 years) were recruited on a voluntary basis. Written informed consent was obtained from each of the participants. A total of 41 patients completed the 2-year follow-up; 4 patients were lost to follow-up (3 in the MMED group and 1 in the MMED+AFS group). No death case was found; a detailed flow chart is showed in Figure 1. The demographic data of the patients is shown in Table 1. No significant differences were identified in the distribution range of patient age, intraoperative blood loss, or length of hospital stay, among all groups.

### 3.2. Improvement of Pain and Disability (VAS, ODI, and SF-36)

**Figure 3:**
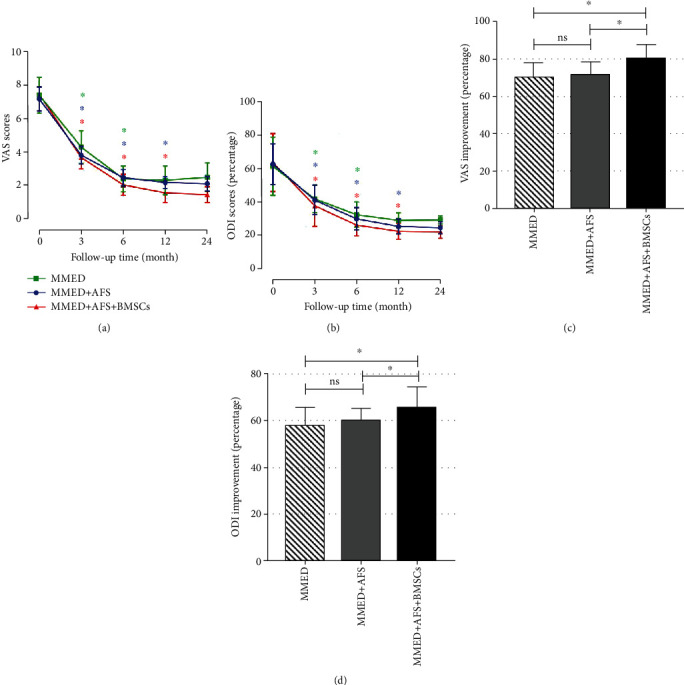
VAS (a) and ODI (b) scores at each visit presented an obvious reduction compared with the preoperative level. The asterisk (∗) indicates that the data at this follow-up time point is statistically significant compared with the data at the previous follow-up time points. At the last follow-up compared with the preoperational status, VAS improvement rate (c) showed that the MMED+AFS+BMSCs group had better pain relief than the MMED and MMED+AFS groups (^∗^*P* < 0.05). In ODI improvement (d), the MMED+AFS+BMSCs group's disability improvement was significantly higher than the other groups (^∗^*P* < 0.05), while the MMED+AFS group showed no significant difference compared with the MMED group (ns; *P* > 0.05). ns: nonsignificant.

**Figure 4:**
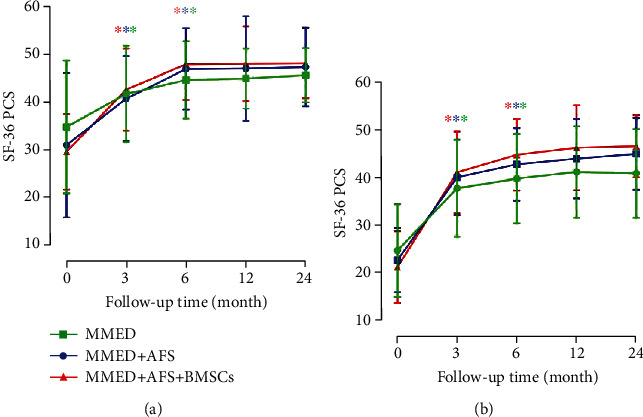
SF-36 of PCS scores (a) and MCS scores (b) showed that all patients presented an improvement of more than 50% in the last follow-up period compared to preoperative status (^∗^*P* < 0.05). No significant difference was found between all groups at the last follow-up (*P* > 0.05). The asterisk (∗) indicates that the data at this follow-up time point is statistically significant compared with the data at the previous follow-up time points.

The mean preoperative VAS score was 7.4 ± 1.1, 7.1 ± 0.7, and 7.4 ± 1.0 in the MMED, MMED+AFS, and MMED+AFS+BMSCs groups, respectively, whereas the mean ODI score was 61.2% ± 17.4%, 62.4% ± 12.2%, and 63.3% ± 14.3%, respectively. All patients achieved significant improvement at 3 months postoperative, and the improvement persisted to the 24-month follow-up (details in Figures 3(a) and 3(b)). At the final follow-up, the VAS improvement rate of the MMED+AFS+BMSCs group significantly improved, compared to the MMED group (80.1% ± 7.6% versus 71.3% ± 7.0%; *P* < 0.05) and the MMED+AFS group (80.1% ± 7.6% versus 70.1% ± 7.8%; *P* < 0.05; details in Figure 3(c)). No significant differences in VAS improvement rates were found between the MMED+AFS group and the MMED group. In addition, the MMED+AFS+BMSCs group also showed an improvement in the ODI changes, compared to the MMED group (65.6% ± 8.8% versus 57.8% ± 8.1%; *P* < 0.05) and the MMED+AFS group (65.6% ± 8.8% versus 59.9% ± 5.5%; *P* < 0.05; details in Figure 3(d)). No significant differences in ODI improvement rates were found between the MMED+AFS group and the MMED group. All patients exhibited a significant improvement in the SF-36 physical score (PCS) or mental score (MCS), and no statistical differences were found between all groups (Figure 4).

### 3.3. Image Analysis

**Figure 5:**
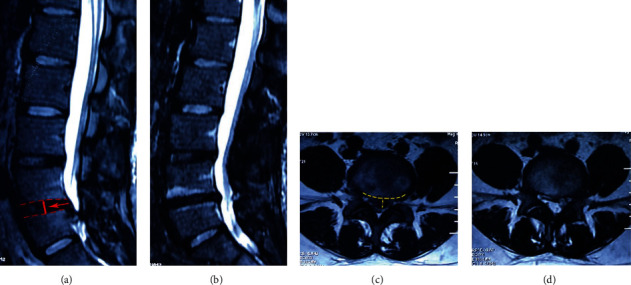
A 41-year-old female, with a chief complaint of low back pain combined with left lower extremity pain and numbness for more than 10 years, failed conservative treatments, and the symptoms progressed in 3 weeks. Preoperative MRI showed a huge herniation bulged into the spinal canal (a, c). She was treated with MMED discectomy combined with BMSC/gelatin sponge composite repairment. MRI scan at 12 months after surgery showed that the Pfirrmann grade of the surgical segment remained at the preoperative level (Grade IV), and the disc protrusion size was significantly decreased, though IDH was slightly decreased (b, d). IDH: intervertebral disc height. The red line in (a) shows the method used to measure IDH; the yellow line in (c) shows the method of measuring disc protrusion size.

MRI changes of a representative patient between the preoperative period and 1 year posttreatment are shown (Figure 5). A 41-year-old female, with a chief complaint of low back pain combined with left lower extremity pain and numbness for more than 10 years, failed conservative treatments, and the symptoms progressed in 3 weeks. Preoperative MRI showed a huge herniation bulged into the spinal canal (Figures 5(a) and 5(c)). After routine examination, she was treated with MMED discectomy combined with BMSC/gelatin sponge composite repairment. MRI scan at 12 months after surgery showed that the Pfirrmann grade of the surgical segment remained at the preoperative level (Grade IV) and the disc protrusion size was significantly decreased, though IDH was slightly decreased (Figures 5(b) and 5(d)).

**Figure 6:**
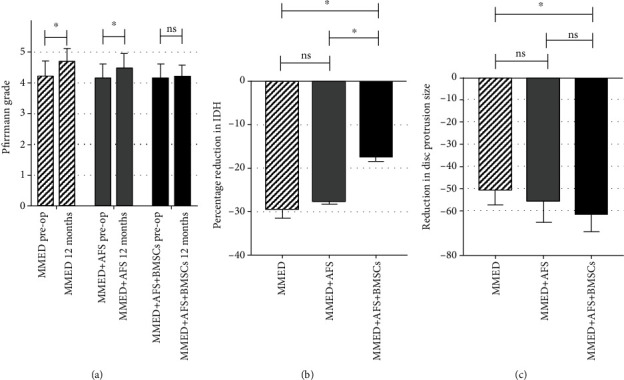
The MMED and MMED+AFS groups' Pfirrmann grade was significantly decreased, while the MMED+AFS+BMSCs group remained at the preoperative level (a). All patients' IDH existed in various degrees of reduction, the MMED and MMED+AFS groups have significant IDH lost at the latest follow-up, and no significant difference was found. The MMED+AFS+BMSCs group presented the fewest IDH lost (b). All patients achieved a reduction in DPS for more than 50% (c). DPS: disc protrusion size; ns: nonsignificant. ^∗^*P* < 0.05.

The level of disc degeneration was measured using MRI and was evaluated using the Pfirrmann classification system. The average baseline level of the Pfirrmann grade is shown in Table 1. At the 12-month follow-up, the mean Pfirrmann grade of the MMED group was significantly increased, compared to preoperative status (4.2 ± 0.5 versus 4.6 ± 0.5; *P* < 0.05), while the MMED+AFS group also observed significant change (4.1 ± 0.4 versus 4.5 ± 0.5; *P* < 0.05). At the final 24-month follow-up, the mean Pfirrmann grade of the MMED+AFS+BMSCs group was not significantly different from the preoperative status (Figure 6(a)). The mean IDH loss rate in the MMED+AFS+BMSCs group (−17.2% ± 1.3%) was significantly lower than the other two groups at 12 months posttreatment, whereas the IDH loss rate of the MMED+AFS group (−27.6% ± 0.7%) has no significant difference than the MMED group (29.3% ± 2.2%; detailed in Figure 6(b)). Based on measurements taken after 12 months postoperative, disc protrusion size (DPS) decreased more than 50% in all patients, compared to preoperative status, and no recurrence of herniation occurred (detailed in Figure 6(c)).

## 4. Discussion

In the past, at the end of every discectomy procedure, the IVD defect was left because of technical difficulties in gaining access. The IVD defects after discectomy were often attributed to the aggravation of IVD degeneration or the recurrence rate of herniation at the same operative segment [1]. In recent years, many researchers have tried to close the IVD defect through AF suture (closure) techniques to restrain degeneration progress, including the use of Xclose and EFit Disposable Annular Stapler device [4–7]. In our clinical department, additional AF sutures had been attempted in hundreds of cases of soft herniation with successful intraoperative closure. According to our application experience, the entire process averaged less than 3 minutes, and no related complications were observed.

Although the safety of the AFS technique has already been confirmed, definite curative effects were still undetermined [4]. Ahlgren et al. first studied the effect of suture repairing AF defect on the healing strength of sheep IVD. After 6 weeks of observation, it was shown that a simple suture of AF defect could not significantly improve the healing of IVD [33]. Sutures cannot compensate for the loss of AF material, nor can they reverse the biomechanical changes that have occurred in the damaged AF [34]. Some scholars hold the view that closing the annulus fissure can promote scar healing and reduce the release of inflammatory mediators in the IVD [35]. In this study, although the VAS, ODI, and SF-36 scores improved in all participants, these differences were not statistically significant between the MMED and MMED+AFS groups. Our results were consistent with a previous report by Bailey et al., whose study showed no significant difference in patient self-reported pain and disability after AF suture using the Xclose Tissue Repair System [4, 7]. In addition, our results showed that the addition of AFS exhibited a relatively weak trend on preventing the degenerative progress, while not statistically significant, as evidenced by maintained IDH and Pfirrmann grading (Figures 6(a) and 6(b)). This was consistent with the findings of a previous animal study, which focused on the specific process of IVD change that after surgical suture of the AF defect, degeneration signs occurred at early 3 weeks, followed by a continuous increase of the Pfirrmann grade through 12 weeks and an obvious loss of the load-barrier ability [36]. Hence, it seems that the simple suture strategy does not have a stable effect on preventing further dehydration and degeneration of IVD, and it cannot reverse the biomechanical changes of IVD.

It is likely that IVD defect repair will be improved by a combination of mechanical and biological strategies. MSCs have been reported in the clinical treatment of IVD degeneration, showing a remarkable potential to repair IVD degeneration [8, 20]. BMA is recognized as having various types of cells and growth factors [28]. Unfortunately, the MSCs represent a very small fraction of about 0.001-0.01% of the nucleated cell count of the BMA, leading to a limited therapeutic effect [25, 27]. The SCR method demonstrates convenience and reliability, with the process of cell isolation taking approximately 5 minutes to form implantable graft materials after a few simple steps. In a clinical report, Yousef et al. had used the Cellect selective cell retention device (Depuy Spine, USA) with a collagen scaffold for posterolateral spinal fusion in twenty-one patients, which augments the spinal fusion rate after a long-term follow-up [25]. In our study, we observed that compared with non-SCR-treated cells, the adhesion rate of SCR-treated MSCs was significantly higher (3.52-fold versus 6.97-fold). In addition, the concentration of various growth factors (BMP-2, IGF-1, and PDGF) produced by the cells also increased significantly (4.47-, 4.72-, and 2.84-fold, respectively). Although the culture-expanded methods increase the number of MSCs by 3,600-fold, two previous animal studies demonstrated that a similar therapeutic effect was observed using either a 3-4 times cell concentration or culture-expanded MSCs [37, 38].

The safety of MSC therapy is controversial which is largely due to the lack of adequate evidence. With the increasing clinical applications of MSCs every year, some graft-related complications have been discovered. These include osteophyte proliferation, heterotopic ossification generated by cell leakage, and neoplastic hyperplasia due to uncontrolled cell pluripotency [39, 40]. Thus, it is important to select an appropriate cell carrier with the ability to prevent cell leakage while providing a suitable environment for MSC growth and differentiation. Gelatin sponges have been widely used in spinal surgery for many years due to their hemostatic properties. Gelatin sponges also have a high degree of histocompatibility, plasticity, and absorbability, which fits the demand for IVD defect repair perfectly [19, 40]. In addition, the high-porosity structure of the gelatin sponge particles is conducive to binding the cells and effective components to the surface of the material during the SCR process. No graft-related complications were found during the 2-year follow-up. Moreover, the additional AF suture may prevent graft leakage. In terms of available evidence, our method is appropriate in both aspects of repairing effect and clinical safety.

There were some limitations in our study. Firstly, patients were not blinded due to the legal restrictions for clinical use of MSCs, which may have resulted in bias in the patient self-report evaluation and scale scoring. In addition, we did not characterize the composition of the enriched BMA. Despite the drawbacks mentioned above, the potential validity of this procedure has been shown in this study, and further randomly controlled prospective trials with a large number of patients and long-term follow-ups will form the focus of future trials.

## 5. Conclusions

Our research showed that it is feasible and effective to repair IVD defects with SCR-enriched BMSCs combined with gelatin sponge followed by AFS, which provides a feasible cell-based therapy for IVD defects after discectomy. Furthermore, although the effect of AFS is uncertain, the closure of AF defect may have assisted in the prevention of cell leakage.

## Figures and Tables

**Table 1:** The demographic and clinical details of the enrolled patients.

Sample size		*N* = 15	*N* = 15	*N* = 15
		MMED	MMED+AFS	MMED+AFS+BMSCs
Age range (year)		23-64 (42 ± 11.5)	28-51 (41 ± 9.7)	25-60 (44 ± 10.7)
Gender	Male	9	11	8
	Female	6	4	7
Operation level	L3-L4	3	2	2
	L4-L5	6	6	4
	L5-S1	6	7	9
Pfirrmann grade (pre)	III	3	2	3
	IV	8	9	9
	V	4	4	3
Operation time (min)		35-50 (40.5 ± 5.8)	40-55 (45.3 ± 5.1)	54-75 (61.2 ± 7.2)
Blood loss (mL)		10-20 (12 ± 3.9)	10-15 (11 ± 4.5)	15-25 (17 ± 4.7)
Hospital stay (day)		8-17 (10 ± 2.3)	8-16 (10 ± 3.1)	6-17 (12 ± 3.9)

## Data Availability

The data used to support the findings of this study are included within the article.

## Supplementary Materials

Supplementary Materials A pilot study based on a goat model was conducted to confirm the effective component in bone marrow aspirate and the enrichment effect of the BONE GROWTH PROMOTER. A brief introduction was described in the supplemental information.
