# Supplementary material for: Selective Retention of Bone Marrow Stromal Cells with Gelatin Sponge for Repair of Intervertebral Disc Defects after Microendoscopic Discectomy: A Prospective Controlled Study and 2-Year Follow-Up
Source: Biomed Res Int. 2021 Jul 13;2021:4822383. doi: 10.1155/2021/4822383 (PMC8294975; doi:10.1155/2021/4822383)
Supplement: Supplementary Materials — A pilot study based on a goat model was conducted to confirm the effective component in bone marrow aspirate and the enrichment effect of the BONE GROWTH PROMOTER. A brief introduction was described in the supplemental information. [file 4822383.f1.docx]

Supplemental Material

In order to study the bone marrow aspirate and enrichment effect, a pilot study was conducted in a goat model. The BMA was aspirated from iliac crest of the goat using a 16 G bone marrow puncture needle. Then, a total of 20 mL BMA aspirated from one side iliac crest was injected into the BGP for enrichment (Fig. S1 A). Six cycles were processed until MSCs and effective constituent in BMA were fully filtered and seeded into the gelatin sponge cubes. The enriched BMA-matrix was taken out and fixed in 4 % paraformaldehyde for 12 h. After dehydration and drying, the sample was cut into 1 mm slices, sprayed with gold and observed by scanning electron microscope.

To assess the enrichment effect, the volume of BMA was measured before and after enrichment process, and the samples were taken for flow cytometry to detect the number of nucleated cells and the number of the target cells with negative expression of CD34 and CD45 and positive expression of CD90. The number and concentration of cells in the BMA before and after BMA enrichment using BGP were obtained by flow cytometry. Then the concentration and adhesion multiple of the retention cells in the gelatin sponge matrix were calculated according to the calculation formula as follows: retention cells concentration= retention cells number/volume of retention fluid; cells adhesion multiple= the retention cells concentration/original BMA cells concentration

Furthermore, 100 μL original BMA and a small amount of enriched matrix were cultured in a culture dish respectively with 10 mL of the DMEM/F12 culture medium containing 10 % FBS for 11 days. Then, the cells adhered to the culture plate surface were identiﬁed by crystal violet stanning to assess the enrichment effect, followed by observation under microscope.


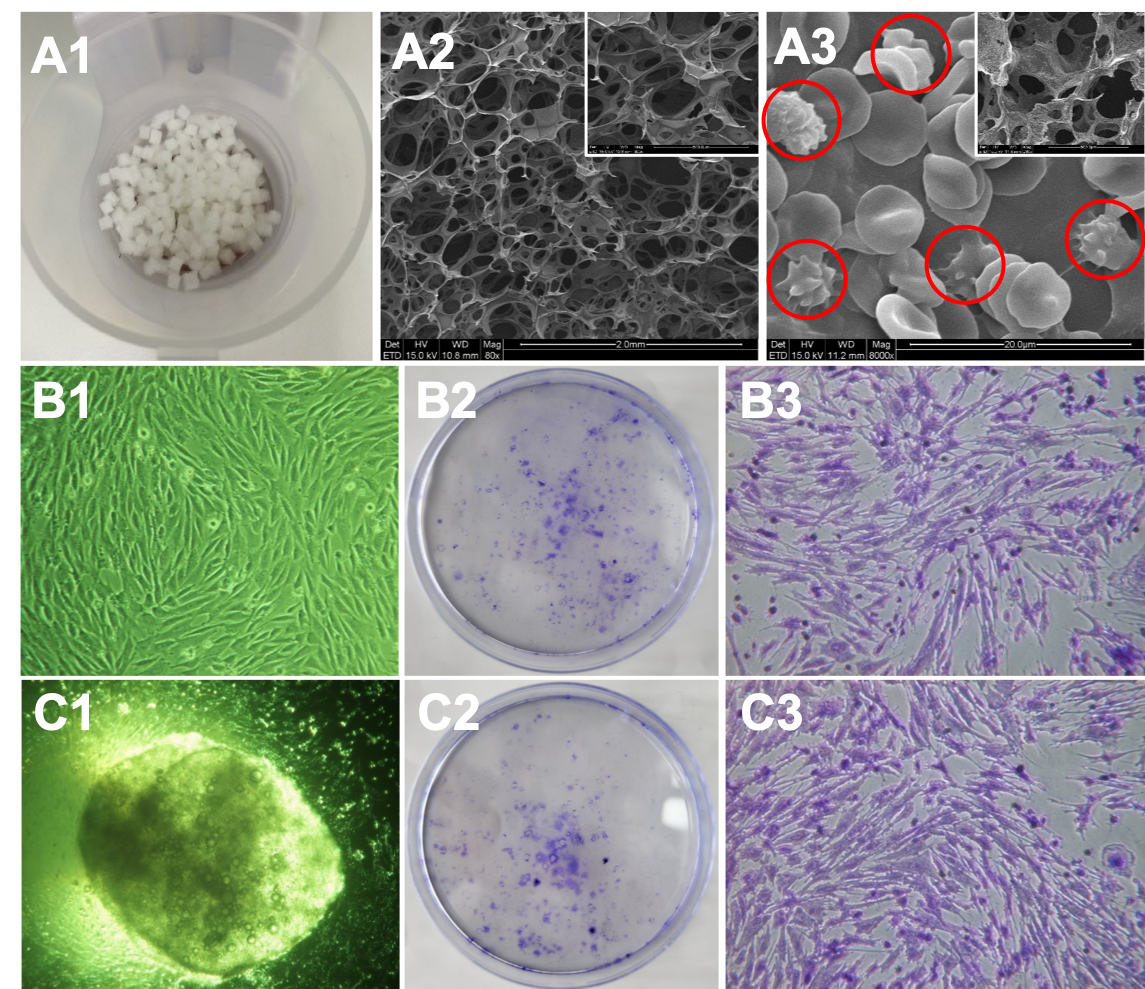


**Fig.S1 Bone marrow enrichment.** (A) Macro and microstructure of gelatin sponge. A1. Gelatin sponge cubes (5 mm × 5 mm × 2.5 mm) in the BGP.A2. SEM images shows the porous structure and their interconnection of gelatin sponge. A3. Multiple round cells adhered to the inner wall of the enriched matrix. (B) The cell colony crystal violet staining of the BMA and the enriched matrix. B1. After the BMA culturing for 11 days, the fibroblastic colony formation could be observed. d. After the enriched BMA-matrix culturing for 11 days, a lot of cells were visible in and around the gelatin sponge. b, e. Many colony-forming units stained by crystal violet in the culture dish of the BMA and the enriched matrix respectively. c f. Microscopic observation showed that a number of fusiform or polygonal cells stained by crystal violet were adherent to the bottom of culture dish from the BMA and the enriched matrix. respectively (×100).


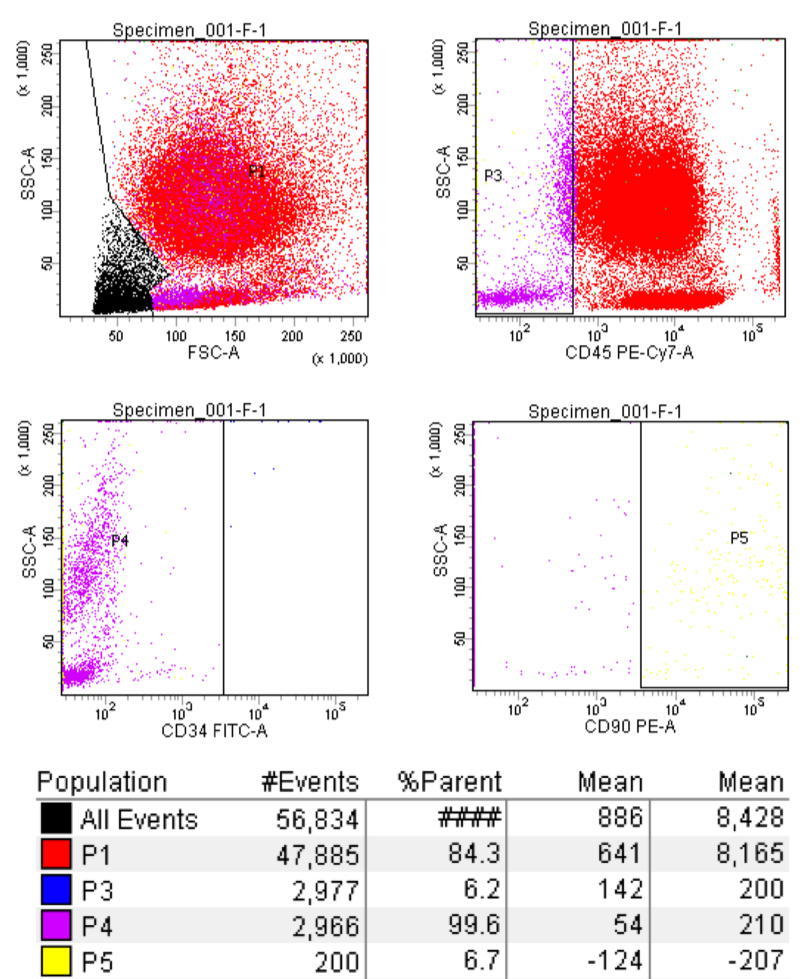


**Fig.S2** **Enrichment effect of BGP.** The number of nucleated cells and target cells in the sample were detected by flow cytometry to calculate the adhesion multiple of the BGP. Calculation formula: adhesion multiple α = cells concentration of retentate/cells concentration of BMA. After the BMA enriched by the BGP, the adhesion times of the retained nucleated cells and the target cells were 6.40 ± 0.93 and 4.20 ± 0.65 respectively.


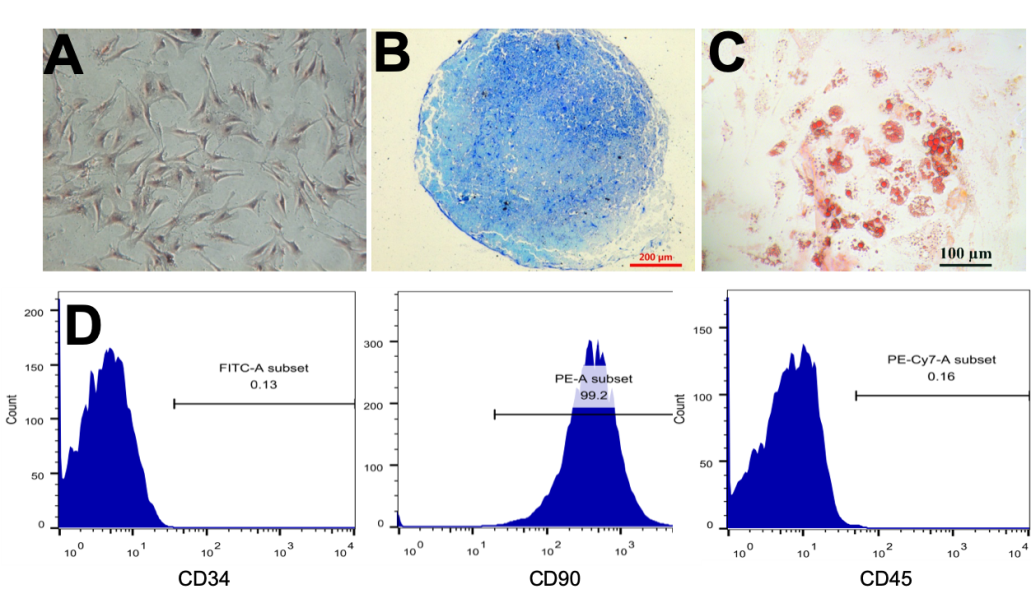


**Fig.S3 Identification of BMSCs.** (a) BMSCs were ALP stained after osteogenesis induction. (b) BMSCs were stained with toluidine blue after chondrogenesis induction. (c) BMSCs were stained with oil red O after fat induction. (d) The CD90 was positively expressed, and CD34 and CD45 were negatively expressed on the surface of the passage 3 BMSCs, which was detected by flow cytometry.
